# Seasonal and Daily Patterns in Activity of the Western Drywood Termite, *Incisitermes minor* (Hagen)

**DOI:** 10.3390/insects2040555

**Published:** 2011-12-12

**Authors:** Vernard Lewis, Shawn Leighton, Robin Tabuchi, Michael Haverty

**Affiliations:** Environmental Science, Policy, and Management, College of Natural Resources, University of California, Berkeley, CA 94720, USA; E-Mails: sleighton@berkeley.edu (S.L.); rtabuchi@berkeley.edu (R.T.); mhaverty@berkeley.edu (M.H.)

**Keywords:** feeding periodicity, seasonality, drywood termites, Kalotermitidae, Isoptera

## Abstract

Activity of colonies of the western drywood termite, *Incisitermes minor*, was measured with acoustic emission (AE) technology in five loquat (*Eriobotrya japonica*) logs. Termite activity, whether it was feeding, excavation or movement, was monitored for 11 months under ambient conditions in a small wooden structure maintained at the University of California Richmond Field Station. AE, temperature, and humidity data were measured in 3-minute increments. Termite activity was greater during the warmer summer months compared to the cooler winter months. Termites in all five logs displayed a similar daily cycle of activity, peaking in the late afternoon. Seasonal and daily fluctuations in termite activity were significantly associated with temperature, whereas humidity did not appear to have a noticeable effect on termite activity. Possible mechanisms that drive the seasonal and daily cycles in termite activity, as measured by AE technology, and the possible implications for inspections and post-treatment analysis are discussed.

## 1. Introduction

Seasonal activity patterns of drywood termites have an important impact on our ability to detect these structural pests and treat infestations. For example, the presence of alates and shed wings within infested structures are often the first signs of the presence of drywood termites. In California, the annual dispersal flights of *Incisitermes minor* (Hagen) occur during the day in the fall [1]. Feeding and foraging, including excavation of wood, are important drywood termite activities; however, little is known whether they occur randomly or follow an underlying pattern. The cryptic behavior of drywood termites hinders studies on their feeding and foraging biology. Because direct observation is impossible in wood naturally infested by drywood termites, we must rely on indirect methods of observation. A method that has proven extremely useful is the quantification of vibrations within termite-infested wood using acoustic emission (AE) technology.

Drywood termites produce vibrations in wood. Some of these vibrations can be heard by the human ear without amplification, but usually require some sort of augmentation [2-6]. These detectable sounds are produced during feeding and foraging activities [7-11] and by vibratory movements (head-banging) of workers and soldiers [12-16]. Acoustic emission devices are available that can successfully detect the vibrations made by drywood termites in laboratory settings at least 80% of the time [10,13,17-22]. Detection of drywood termite activity has been reported from 80 cm to 240 cm along the length and <8 cm across the grain of a board in relation to the location of live termites [10,22].

These devices display values that represent termite activity as either AE events or AE ring down counts. AE events are defined as “a microstructural displacement that produces elastic waves in a material under load or stress” [23]. An AE event is thought to be the actual pulling of wood fibers by termites and by the movement of termites within the wood [10,13,17]. AE ring down counts refer to the propagation of AE-generated feeding energy throughout the board [24]. A homologous example of these variables can be thought of as a raindrop hitting a pond (event) and similarly the wave propagation amplitude throughout the pond being the AE ring down count.

AE events and AE ring down counts are good measures of the presence or absence of termites (80%); however, Sheffrahn *et al.* [10] demonstrated that they are only moderately associated (r^2^ = 0.45) with the number of termites within infested wood at the time of recording. Using AE technology to record feeding by *I. minor*, Lemaster *et al.* [22] found no specific pattern in feeding activity over a 24-hour period. However, the investigation only ran for one week. Indrayani *et al.* [25] also used AE technology to monitor feeding of laboratory groups of *I. minor* when exposed to different temperatures and relative humidities; however, their study was not designed to evaluate seasonal or daily variation in termite activity. During a field investigation in southern California using AE technology for monitoring the effectiveness of local chemical treatments, V. Lewis [unpublished] found that activity of *I. minor* colonies in untreated locations of structures declined during winter months. Because this study involved only four post-treatment inspection dates, it is impossible to make definitive statements on seasonal feeding or foraging activity of *I. minor*.

Little research has been conducted on the movement patterns of drywood termites. Currently, only the speed of locomotion of *I. minor* (1.4 cm/s) in response to temperature and light is known [26,27]. Movement of the drywood termite *I. fruticavus* Rust was inferred from studies that measured daily changes in temperature inside galleries for the Jojoba shrub, *Simmondsia chinensis* (Link) [28]; however, the seasonal movement of drywood termites within structures remains poorly understood.

The purpose of our study was to explore for patterns of seasonal or daily feeding and movement of *I. minor* in naturally infested logs as measured by AE technology. Such knowledge would greatlyimprove inspection success, pre- and post-treatment evaluations of remedial measures againstinfestations and enable more detailed investigations into the natural history of drywood termites.

## 2. Experimental Section

### 2.1. Preparation and Selection of Naturally Infested Logs

Seven logs from a large loquat (*Eriobotrya japonica* (Thunb.) Lindl.) tree were collected from a private residence in southern California (Granada Hills, CA, USA) The logs were similar in maximum diameter, length, and age. To verify candidate logs were active with drywood termites, using the methods from Lewis and Haverty [21], three 1-min recordings from the midpoint on the topside of the log were taken using a hand-held device (Tracker, Dunegan Engineering, Midland, TX, USA). All logs having at least 300 counts per minute were chosen to be included in the study. Five logs containing termites were selected to record AE activity and two logs containing no termites were chosen as controls to measure background AE activity from the surroundings.

The two control logs were placed into an oven (Isotemp model 655F, Fisher Scientific, Pittsburgh, PA, USA) at 105 °C for three days to kill any termites present in them. All seven logs had a subsurface sensor installed into their long center by drilling a 2.4-mm diameter hole and inserting the sensor probe 1.2 cm deep into wood. All log and sensor assignments were chosen randomly. A 3-m long cable from each of the seven sensors was connected into a port in the back of an AE smart device [29] and dedicated computer (Dell Corporation, Austin, TX, USA) that stored all of the data. The custom software used provided for twenty 3-minute recordings that were taken randomly among the seven sensors for each 60-minute period during the study. No sensor was used more than three times or less than two times during a 60-minute period, and the order of the readings was randomized. In addition, temperature and humidity (Omega Engineering, Stamford, CT, USA) were recorded with each 3-minute AE recording and saved to an electronic spreadsheet (Excel Corporation, Lubbock, TX, USA). A backup battery power supply (Back-ups, APC Corporate, W. Kingston, RI, USA) was also installed in the event of unexpected power outages. The entire AE data gathering and storage system was run twenty-four hours a day for almost 11 months (15 June 2008 to 15 May 2009). All logs, AE and temperature equipment were housed in a small wooden building at the University of California Richmond Field Station, Richmond, CA, USA. The building had five windows for natural light, and was without air conditioning, heaters, or insulation.

### 2.2. Data Summarization and Display

The hourly averages for AE ring down counts for each active and inactive log, as well as temperature and relative humidity, were plotted for the 11-month period. The mean daily activity pattern for *I. minor* in each of the five logs (sensors 1–5), represented by AE ring down counts, was graphed in relation to the mean temperature (°C) over a 24-hour period.

## 3. Results and Discussion

### 3.1. Seasonal Patterns in AE Activity

**Figure 1:**
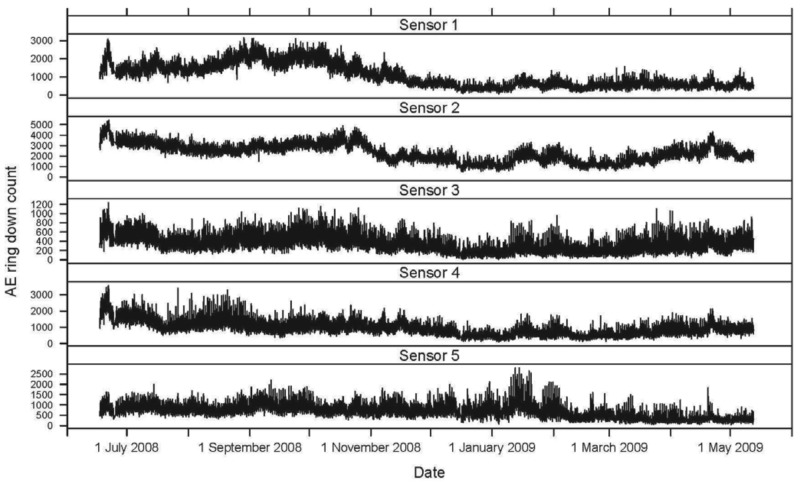
A continuous record of the acoustic emission (AE) ring down count for each sensor (1–5), from 15 June 2008 to 15 May 2009, displays a seasonal cycle. The AE ring down counts for the untreated checks (sensors 6 and 7), logs with no live termites, are displayed for comparison.

**Figure 2:**
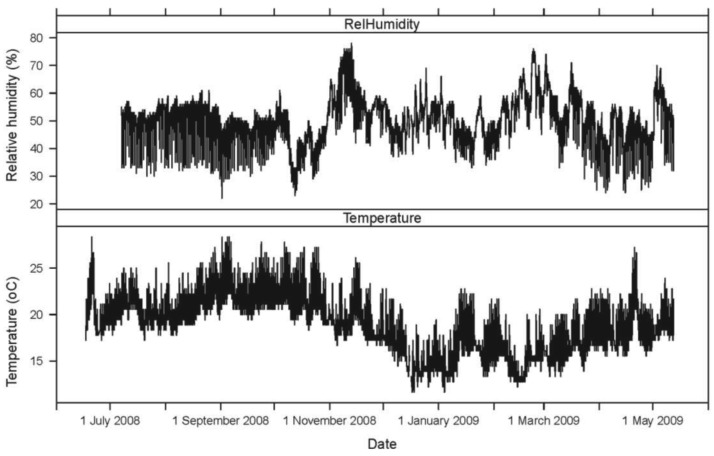
Temperature (°C) and relative humidity (%) traces for the building used to house the AE activity study at the University of California Richmond Field Station from 15 June 2008 to 15 May 2009.

Seasonally, AE ring down counts displayed a non-linear pattern of increasing and decreasing values associated with temperature (Figures 1 and 2). Termite activity was highest during the warmer spring and summer months compared to winter. However, an increase in daytime temperature or a sudden heat wave, even in January and February 2009, resulted in a burst of termite activity (Figures 1 and 2). Humidity did not appear to have a significant impact on termite activity during the 11-month study.

### 3.2. Diurnal Patterns in AE Activity

**Figure 3:**
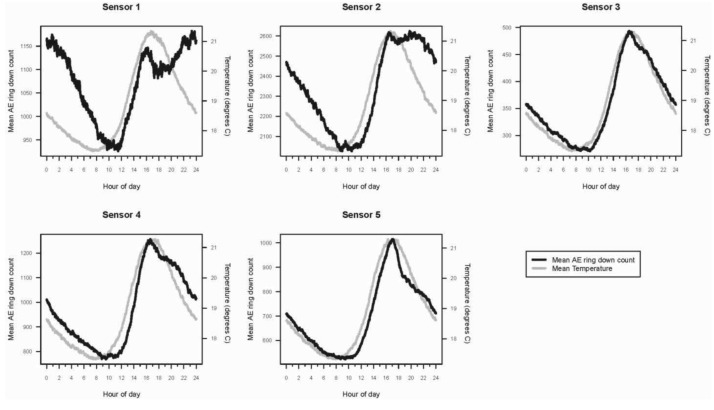
Mean daily activity patterns for *I. minor* in each of five logs (sensors 1–5). The heavy, dark line is the mean AE ring down count. The gray line is the temperature trace (°C) collected simultaneously for each AE data point. AE data for all logs were collected from 15 June 2008 to 15 May 2009.

Within a 24-hour day, AE ring down counts among the logs displayed a non-linear pattern of activity (Figure 3). These patterns were sinusoidal in shape for all sensors. Termite activity was lowest during the morning, increased in the afternoon, and peaked in late afternoon (1800), then declined until mid-morning. Based on the AE activity, the logs appeared to contain termite populations of different sizes: the smallest in log (sensor) 3, roughly three times larger in logs (sensors) 1, 4, and 5, and almost seven times larger in log (sensor) 2. The rise and fall in termite activity was correlated with temperature: activity increased as temperature rose (Figure 3). However, termite activity was apparently not significantly affected by relative humidity.

There are only a few reports available on seasonal or daily activity for *I. minor* as measured by AE technology. Using 100 workers contained in an artificially infested wooden block held at constant temperature and humidity, Lemaster *et al.* [22] presented AE event results over seven days from a single sensor. No statistically significant cycling or periodicity in AE measurements could be detected. The plot of termite activity appeared flat and ranged from just 100 to 200 AE events per hour.

There is considerable variance in the number of *I. minor* extracted from naturally infested logs and structural wood, and ranges from 7 to 2,943 [19,21,30-32]. Also clearly evident, larger colonies and infestations produce greater AE activity. And also clearly evident is that when drywood termites are allowed to search and forage for wood naturally under ambient conditions, their activity follows a cyclic pattern common to many terrestrial animals.

A second termite activity study using AE technology was conducted by Indrayani *et al.* [25] under laboratory conditions. Groups of 10 *I. minor* pseudergates were placed inside small wooden blocks to investigate the effects of varying temperature and humidity. The experiments lasted only 12 hours. The optimum temperature for peak AE activity was 30 °C. Reviewing the temperature and AE data from our study, termite activity increased with temperature, even during warm days during seasonally cold months (November 2008 to February 2009; Figures 1 and 2).

Knowledge on optimal times for drywood termite foraging could be important for termite inspections. Two species, *I. minor* and *Cryptotermes brevis* (Walker), are responsible for a majority of the damage caused by drywood termites in the United States [33-35]. The economic cost of control and repair of damage is second only to that of subterranean termites [34]. Traditional inspections are visual and are based on searches for damaged wood or pellets. The data from this study, as well as that in the Indrayani *et al.* [25] study, suggests searches for termite infestations could be enhanced by heating the wood to at least 25 °C prior to inspection to simulate foraging and feeding, even in winter. A more in-depth understanding the underlying mechanism that controls the cyclic pattern will require additional studies that include the exclusion of natural light, additional tests of naturally infested logs at constant temperature and humidity, and modifying the AE system collection hardware and software to sort out AE events or ring down counts due to the various behaviors including locomotion, feeding and foraging activity, and other vibrational behaviors.

## 4. Conclusions

Five logs (loquat *Eriobotrya japonica*) containing live colonies of the drywood termite *I. minor* were monitored under ambient conditions in a building to measure acoustic emissions as a surrogate for feeding activity, as well as temperature and relative humidity. Over the 11-month observation period, the seasonal and daily feeding activity of *I. minor* followed a sinusoidal curve, with peak feeding activity closely following the raise and fall of temperature. Seasonal feeding activity was greatest during the summer months or periods of very warm days (as high as 23 °C) during winter. Daily feeding patterns were characterized as increasing in the late morning into late afternoon and decreasing in the early evening. Results from this study can be used to determine the optimal times of the day and year to search for drywood termites, especially during winter months when heating suspicious locations can increase the colony activity.
